# Anti-IL1ß-monoclonal antibody in two adult patients with PFAPA Syndrome - a single centre experience

**DOI:** 10.1186/1546-0096-13-S1-P135

**Published:** 2015-09-28

**Authors:** B Kortus-Götze, J Hoyer

**Affiliations:** 1 University of Marburg, Nephrology, Marburg, Germany

## Background

The periodic fever, aphthous stomatitis, pharyngitis and cervical adenitis (PFAPA) syndrome belongs to the rare nonhereditary autoinflammatory diseases and normally onsetting in childhood, but despite of tonsillectomy persisting or newly occurring in adulthood. The exact pathogenesis is not clear, but to our understanding it is an acquired autoinflammatory disease due to an unregulated production of cytokines. The common treatment varies from NSAIDS, colchicine, corticosteroids up to blocking IL1- receptor.

## Objectives

The anti-IL1ß-monoclonal antibody canakinumab has been introduced as a specific therapy in patient with cryopyrin-associated periodic syndromes (CAPS). There is only very few data about the effect of canakinumab in patients with PFAPA syndrome in off label use.

## Methods

Here we report about two unrelated female patients, 20 and 49 years old, with PFAPA syndrome diagnosed in 2014 respectively 2012 with no treatment response to NSAIDS, colchicine, corticosteroids and severe side effects or no reduction of disease activity under the therapy with anakinra. Therefore, we initiated a subcutaneous therapy with canakinumab using standard dosage of 150 mg every eight weeks while monitoring clinical response and inflammation markers C-reactive protein (CRP) plus serumamyloid A (SAA).

## Results

Immediately after first injection both patients showed a good response to canakinumab with reduced activity of inflammation markers, reduction of the typical clinical symptoms and ultimately with an improved quality of life. Even over the period of 6 months respectively two years we have seen a lasting effect of canakinumab with reduction of disease activity.

## Conclusions

In our experience, treatment with the anti-IL1ß-monoclonal antibody canakinumab might be an effective, safe and feasible treatment showing no severe side effects and should be considered as a new therapeutic option in off label use in patients with PFAPA syndrome.

